# Analysis of microRNA from archived formalin-fixed paraffin-embedded specimens of amyotrophic lateral sclerosis

**DOI:** 10.1186/s40478-014-0173-z

**Published:** 2014-12-14

**Authors:** Koichi Wakabayashi, Fumiaki Mori, Akiyoshi Kakita, Hitoshi Takahashi, Jun Utsumi, Hidenao Sasaki

**Affiliations:** Department of Neuropathology, Institute of Brain Science, Hirosaki University Graduate School of Medicine, 5 Zaifu-cho, Hirosaki, 036-8562 Japan; Department of Pathological Neuroscience, Center for Bioresource-based Researches, Brain Research Institute, University of Niigata, Niigata, Japan; Department of Pathology, Brain Research Institute, University of Niigata, Niigata, Japan; Department of Neurology, Hokkaido University Graduate School of Medicine, Sapporo, Japan

**Keywords:** AMBRA1, Amyotrophic lateral sclerosis, Autophagy, Bioinformatics, Formalin-fixed paraffin-embedded specimen, MicroRNA

## Abstract

**Background:**

MicroRNAs (miRNAs) are noncoding small RNAs that regulate gene expression. This study investigated whether formalin-fixed paraffin-embedded (FFPE) specimens from postmortem cases of neurodegenerative disorders would be suitable for miRNA profiling.

**Results:**

Ten FFPE samples from 6 cases of amyotrophic lateral sclerosis (ALS) and 4 neurologically normal controls were selected for miRNA analysis on the basis of the following criteria for RNA quality: (i) a postmortem interval of less than 6 hours, (ii) a formalin fixation time of less than 4 weeks, (iii) an RNA yield per sample of more than 500 ng, and (iv) sufficient quality of the RNA agarose gel image. An overall RNA extraction success rate was 46.2%. For ALS, a total of 364 miRNAs were identified in the motor cortex, 91 being up-regulated and 233 down-regulated. Target genes were predicted using miRNA bioinformatics software, and the data applied to ontology analysis. This indicated that one of the miRNAs up-regulated in ALS (miR-338-3p) had already been identified in leukocytes, serum, cerebrospinal fluid and frozen spinal cord from ALS patients.

**Conclusion:**

Although analysis was possible for just under half of the specimens examined, we were able to show that informative miRNA data can be derived from archived FFPE samples from postmortem cases of neurodegenerative disorders.

**Electronic supplementary material:**

The online version of this article (doi:10.1186/s40478-014-0173-z) contains supplementary material, which is available to authorized users.

## Introduction

MicroRNAs (miRNAs) are small, single-stranded, noncoding RNAs that regulate gene expression at the transcriptional and translational levels in both plants and animals [1]. A single miRNA may bind to as many as 200 target genes [2]. miRNAs are of great interest because they can regulate approximately 30% of human genes [3] and have a huge impact on a wide range of basic biological processes including developmental timing, cell death, cell proliferation, hematopoiesis and patterning of the nervous system [4]. The implications of miRNA network dysregulation have already been well demonstrated in the field of cancer research, suggesting that miRNAs may be good biomarkers for cancer diagnosis and prognosis [5]. The role of miRNAs has also been studied in neurodegenerative conditions such as Alzheimer’s disease [6-22], Parkinson’s disease [19,23-27], Huntington’s disease [28-30], multiple system atrophy [31] and amyotrophic lateral sclerosis (ALS) [19,32-36], postmortem frozen brain tissue having been employed in most cases [6-22,26,28,30,31]. Several investigators have also analyzed miRNAs from cerebrospinal fluid (CSF) [6,34,37], peripheral blood [33,34,37-39] and skeletal muscle [32,35].

Although the yield, quality and integrity of RNA can be reduced through cross-linking with proteins, enzyme degradation as well as chemical degradation during the fixation process [40-44], the expression of miRNAs in formalin-fixed paraffin-embedded (FFPE) samples is known to be well correlated with that in fresh frozen samples [45]. Moreover, the expression of miRNAs is preserved after routine fixation in formalin (up to 5 days) and long-term storage in paraffin (up to 10 years) [45]. Therefore, FFPE samples have recently been used for studies of miRNA in cancer [46-51]. However, the stability and expression of miRNAs in FFPE specimens obtained postmortem and fixed for longer time periods (weeks or months) have not been investigated.

In the present study, we isolated RNAs from archived FFPE brain specimens of postmortem cases of ALS and neurologically normal controls. Although miRNA analysis was possible for only a minority of FFPE blocks, we were able to show that informative data can be derived from selected FFPE postmortem specimens of human brain.

## Materials and methods

### Subjects

To investigate the effect of postmortem interval, formalin fixation and storage period on the stability of RNA, we selected 10 samples (Table 1) that had been obtained at autopsy from 1 to 10 hours after death. The brains from which the specimens had been obtained had been immersed in 10% or 20% formalin or 10% phosphate-buffered formalin for 3–16 weeks. After fixation, the cerebrum had been cut into slices 10 mm thick in the coronal plane. Samples had then been removed from each slice and subjected to tissue processing (dehydration, clearing and impregnation) on an automated instrument (Tissue-Tek VIP 5 Jr., Sakura Finetek Japan, Tokyo, Japan) that employed seven steps of 100% ethanol, three steps of xylene, and four steps of paraffin, with 8 hours at each step. The instrument was operated under vacuum and heated to 37°C for the ethanol and xylene steps and 60°C for the paraffin steps. After tissue processing, each specimen had been embedded in paraffin, and the paraffin blocks had been stored for 10–86 months at room temperature protected from air and sunlight.

**Table 1:** **Summary of fixed paraffin-embedded samples used for RNA isolation**

**Sample no.**	**Pathological diagnosis**	**Postmortem interval (hours)**	**Fixation time (weeks)**	**Fixative**	**Storage period (months)**	**Sample size (mm)**	**RNA yield (ng)**
1	ALS	1	4	20% F	24	30×40	3198
2	ALS	2	4	10% BF	49	30×40	4178
3	ALS	9	8	20% F	56	30×50	911
4	ALS	9	12	20% F	10	30×50	601
5	ALS	9	16	20% F	86	25×35	515
6	ALS	4	9	10% BF	24	20×25	594
7	ALS	4	10	10% BF	12	20×20	251
8	Control	4	4	10% F	38	30×40	2769
9	Control	10	3	10% F	86	25×40	1414
10	Control	10	4	10% F	64	30×45	765

ALS, amyotrophic lateral sclerosis, F, formalin, BF, buffered formalin.

On the basis of the criteria reported by Osawa et al. [50], four criteria were adopted for establishing the suitability of the samples for RNA analysis: (i) a postmortem interval of less than 6 hours, (ii) a formalin fixation time of less than 4 weeks, (iii) a total RNA yield per sample of more than 500 ng, and (iv) sufficient quality of the RNA electrophoresis pattern.

We further evaluated an additional 16 samples (8 cases of ALS and 8 cases of normal controls) for which the postmortem interval had been less than 6 hours and the formalin fixation time had been less than 4 weeks. Thus, we evaluated a total of 26 samples. On this basis, 10 FFPE samples were selected for miRNA analysis, comprising 6 cases of sporadic ALS and 4 neurologically normal controls (Table 2). The FFPE specimens employed were from the motor cortex of patients with ALS and normal subjects. All the diagnoses had been confirmed by neuropathological examination using immunohistochemistry for TDP-43 and ubiquitin. This study was approved by the Institutional Ethics Committee of Hirosaki University Graduate School of Medicine, Japan.

**Table 2:** **Characteristics of postmortem cases in microRNA study**

**Case no.**	**Pathological diagnosis**	**Age/gender**	**Disease duration (months)**	**Postmortem interval (hours)**	**Fixation time (weeks)**	**Storage period (months)**
1	ALS	68/M	12	1	4	24
2	ALS	60/M	108	2	4	49
3	ALS	59/M	36	9	8	56
4	ALS	75/M	11	4	4	36
5	ALS	73/M	9	1.5	4	60
6	ALS	72/M	120	2	4	60
7	Control	67/F		4	4	38
8	Control	71/F		10	3	86
9	Control	84/M		10	4	64
10	Control	60/F		2	4	12

### RNA extraction

Two 5-μm-thick sections were cut from each block and placed in sterile 1.5-mL centrifuge tubes ready for extraction. Tubes containing cut FFPE sections for RNA purification were stored at −80°C until use. Total RNA including small RNAs was extracted using an Arcturus® Paradise® PLUS FFPE RNA Isolation Kit (Life Technologies Corporation, Carlsbad, CA, USA) with the following modifications. Paraffin was removed by xylene treatment and the tissues were washed with ethanol twice to remove the xylene. The tissues were then treated with proteinase K at 37°C overnight, as proteinase K enables extraction of almost the same amount of RNA from FFPE specimens as from fresh frozen samples [52]. After centrifugation, the supernatant was processed with a silica-based spin column (Toray Industries Inc., Tokyo, Japan) in order to obtain purified total RNA. The degrees of RNA cross-linking and RNA degradation were analyzed by agarose gel electrophoresis using an Agilent 2100 Bioanalyzer (Agilent Technologies, Santa Clara, CA, USA). RNA yield was determined from the *A*_260_/*A*_280_ absorbance ratio using a NanoDrop ND-1000 spectrophotometer (Thermo Fisher Scientific, Waltham, MA, USA).

To assess the feasibility of analyzing RNA extracted from FFPE samples, we applied the selection criteria for RNA quality reported by Osawa et al. [50], as described above. The RNA electrophoresis pattern was found to be crucial for estimation of RNA quality. When the majority of RNAs were >4000 nucleotides (nt) in size due to cross-linking or when almost all of the RNAs were fragmented (e.g., <1000 nt), the RNA quality was considered unsuitable for miRNA analysis.

### miRNA expression profiling

Extracted samples of total RNA that satisfied our criteria were labeled with Hy5 using a miRCURY LNA Array miR labeling kit (Exiqon, Vedbaek, Denmark). The labeled RNAs were then hybridized onto a 3D-Gene human miRNA oligo chip (Toray Industries Inc.). The annotation and oligonucleotide sequences of the probes conformed to the miRBase miRNA database Release 17v1.0.0 (http://www.mirbase.org/). After stringent washing, the fluorescent signals were scanned with a 3D-Gene Scanner (Toray Industries Inc.) and analyzed using 3D-Gene Extraction software (Toray Industries Inc.).

The raw data for each spot were normalized by subtraction of the mean intensity of the background signal determined from the signal intensities of all blank spots with 95% confidence intervals. Measurements for spots with signal intensities greater than 2 standard deviations (SD) of the background signal intensity were considered to be valid. The relative level of expression for a given miRNA was calculated by comparing the signal intensities of the valid spots throughout the microarray experiments. The normalized data were globally normalized per array, adjusting the median of the signal intensity to 25.

Any signal intensity level over 50 was considered to be significant. The signal was considered to be up-regulated when log_2_X was increased by 0.1 or more (≧log_2_X = +0.1) compared with the control signal level, and down-regulated when log_2_X was decreased by −0.1 or less (≦log_2_X = −0.1) compared with the control signal level.

### miRNA targets and pathway analysis

Bioinformatics prediction of target genes and miRNA binding sites was performed using miRmap web-based open source software (http://mirmap.ezlab.org/) [53]. Canonical function and ontology analyses for candidate miRNA targets were performed using MetaCore Functional Analysis (ver.6.19, Thomson Reuter/GeneGo, http://lsresearch.thomsonreuters.com/) which is an integrated knowledge base and pathway analysis tool based on a proprietary manually curated database of human protein–protein, protein–DNA and protein compound interactions, and metabolic and signaling pathways, all supported by proprietary ontologies. Gene ontology enrichment analysis was also performed with Gene Ontology Consortium (GOC) web tool (http://geneontology.org/) to confirm standard GO term on the coincidences.

### Immunohistochemistry

Since *SOGA1* (suppressor of glucose by autophagy) [54] was identified as one of the candidate target genes of both up-regulated and down-regulated miRNAs, we evaluated the protein expression levels of autophagy-related genes such as AMBRA1 [55], Beclin 1 [56], ULK1 [57] and ULK2 [57] using immunohistochemistry. Serial 4-μm-thick FFPE sections from the motor cortex and spinal cord (7th cervical, 8th thoracic and 4th lumbar segments) of ALS patients (n = 13) were employed. We also examined neurologically normal individuals (normal control) (n = 6) and patients with various neurological diseases affecting the spinal anterior horn (diseased control) (n = 6). Three sections 40 μm apart were subjected to immunohistochemistry using the avidin-biotin-peroxidase complex method with a Vectastain ABC kit (Vector, Burlingame, CA, USA). The sections were subjected to heat retrieval using an autoclave for 10 min at 121°C in 10 mM citrate buffer (pH 6.0), and then immunostained with rabbit polyclonal antibodies against AMBRA1 (NOVUS USA, Littleton, CO, USA; 1:1000), Beclin 1 (NOVUS USA; 1:200), ULK1 (Thermo Scientific, Rockford, IL, USA; 1:100) and ULK2 (Thermo Scientific; 1:500). Diaminobenzidine was used as the chromogen, and the sections were counterstained with hematoxylin.

### Semi-quantitative analysis

Since AMBRA1 immunoreactivity was decreased in the spinal anterior horn cells in ALS, we assessed the number of AMBRA1-immunoreactive neurons in the spinal anterior horn of control subjects and patients with ALS using a semi-quantitative rating scale, as reported previously [58]: −, unstained; +, weakly stained; ++, moderately or intensely stained. In each case, the numbers of neurons were counted in Rexed’s laminae VIII and IX of the lumbar spinal cord. Counting was performed at an original magnification of x200 using an eyepiece graticule and parallel sweeps of the microscope stage.

### Statistical analysis

Calculations were performed using Statcel software (OMS Publishing, Tokorozawa, Japan). Repeated measures analysis of variance and Student’s or Welch’s *t* test were used to evaluate possible differences in staining intensity between normal controls, diseased controls and ALS. Values were expressed as mean ± standard error of the mean. Correlations at p < 0.05 were considered to be significant.

## Results

### Stability of RNA in postmortem samples

**Figure 1:**
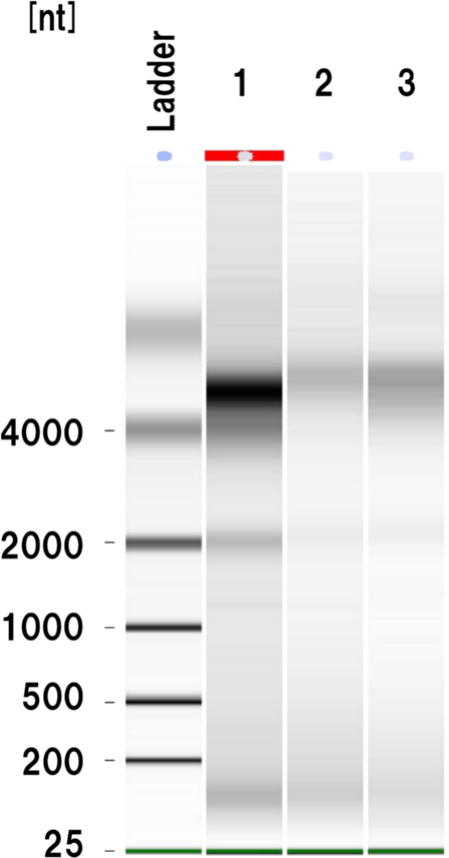
**Effect of formalin fixation on RNA expression.** Representative RNA agarose gel image showing a band of 2000 nt corresponding to 18S ribosomal RNA in lane 1. A faint band of 2000 nt is also seen in lanes 2 and 3. Lane 1, sample 2; lane 2, sample 1; lane 3, sample 8.

The RNA yield was not correlated with the period of storage of FFPE blocks. However, the RNA yield was influenced by the period of formalin fixation (Table 1), being significantly higher in samples that had been fixed for a short period (3–4 weeks; mean 2465 ng) than in those that have been fixed for a long period (8–16 weeks; mean 574 ng) (p < 0.05). The RNA yield did not appear to be affected by the type of fixative employed. Postmortem interval could be also relevant. Samples with higher RNA yield (samples 1, 2 and 8) were the cases in whom postmortem interval was within 4 hours. The rest with lower RNA yield (samples 3–7, 9 and 10) had a combination of longer postmortem interval (9–10 hours) and/or fixation (8–16 weeks). RNA integrity was checked by electrophoresis, and a representative RNA agarose gel image is shown in Figure 1. A band of 5000 nt corresponding to 28S ribosomal RNA was slightly shifted toward a higher molecular weight in all cases, indicating overfixation with formalin and the presence of chemical modifications of the RNA such as covalently linked residual amino acids [59]. A band of 2000 nt corresponding to 18S ribosomal RNA was seen in some cases (Figure 1), suggesting that the extracted RNA was of good quality. These bands were not only shifted, but also appeared more diffuse and less focused. The bands of 100–200 nt corresponded to degraded RNA, as reported previously [60]. On the basis of these initial analyses, we adopted four criteria to indicate that RNA would be of sufficient quality for analysis, based on Osawa et al. [50]: (i) a postmortem interval of less than 6 hours, (ii) a formalin fixation time of less than 4 weeks, (iii) a total RNA yield per sample of more than 500 ng, and (iv) a RNA electrophoresis pattern of good quality.

We evaluated an additional 16 samples (8 cases of ALS and 8 cases of normal controls) for which the postmortem interval had been less than 6 hours and the formalin fixation time had been less than 4 weeks. The RNA yield was more than 500 ng for 12 of these samples, and the RNA agarose gel image was of sufficient quality in 4. As a result, we evaluated a total of 26 samples. The RNA yield was more than 500 ng in 20 of the 26 samples, and the RNA agarose gel image was of sufficient quality in 12. Thus, the success rate for analysis of RNA from FFPE samples of the human postmortem brain was 46.2% (12 of 26).

Based on the above step, 10 cases were selected for miRNA analysis; these included cases of sporadic ALS (n = 6) and neurologically normal controls (n = 4) (Table 2). In case 3, although the fixation time had been 8 weeks, it was included in the present analysis, because the RNA yield was more than 500 ng and the RNA agarose gel image was of sufficient quality.

### miRNA analysis and candidate target genes in ALS

A total of 364 miRNAs were isolated from the motor cortex of patients with ALS. Forty miRNAs showed no change (−0.1 < log_2_X < +0.1), and 91 were up-regulated (log_2_X≧ + 0.1) and 233 were down-regulated (log_2_X≦ − 0.1) relative to the controls. Top 20 microRNAs up- or down-regulated in ALS are shown in Additional file 1: Table S1. miR-494 was found to be the most highly differentially up-regulated miRNA (+4.99-fold change), followed in order by miR-4257, miR-24-3p, miR-4299, and miR-1973 as the top 5 up-regulated miRNAs. On the other hand, miR-4740-5p (+0.19-fold change), miR-1290, miR-3619-3p, miR-1246, and miR-3180-3p were the top 5 down-regulated miRNAs.

**Figure 2:**
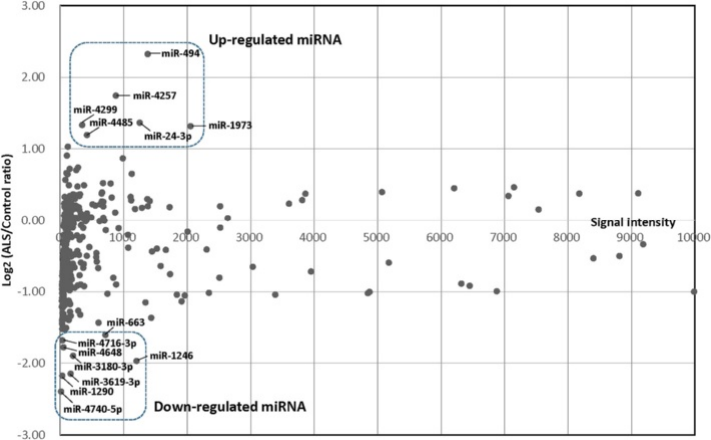
**Scatter plot of all 364 miRNAs comparing signal intensity (X-axis) versus log**_**2**_ **fold-change of amyotrophic lateral sclerosis (ALS)/control ratio (Y-axis).** Based on the size of variation, 6 up-regulated and 8 down-regulated miRNAs were selected in ALS.

Scatter plot of all 364 miRNAs comparing signal intensity versus log_2_ fold-change of ALS/control ratio is shown in Figure 2. We selected 6 up-regulated miRNAs (miR-494, miR-4257, miR-24-3p, miR-4299, miR-1973 and miR-4485) and 8 down-regulated miRNAs (miR-4740-5p, miR-1290, miR-3619-3p, miR-1246, miR-3180-3p, miR-4648, miR-4716-3p and miR-663) in ALS. The candidate target genes of the 6 up-regulated and 8 down-regulated miRNAs in ALS were identified using miRmap web-based open source software.

Top 50 candidate target genes of each of the 6 up-regulated miRNAs in ALS were selected according to the miRmap score. Of the 300 target candidates identified for each of the 6 miRNAs, there was an overlap of 13 genes (Additional file 2: Table S2). These 13 genes have been reported to be involved in muscular cell proliferation (FOXK1 [61], MEF2D [62]), synaptic transmission (ITSN1 [63], RAB3B [64], SLC6A5 [65]), mitochondrial regulation (IBA57 [66], PPARGC1B [67]) and autophagy (MEF2D [68], SOGA1 [54]). These genes may be down-regulated by the 6 up-regulated miRNAs in affected brain regions of ALS. In addition, miR-24-3p showed the most matching frequency (7 overlapped in 50 targets) in up-regulated miRNAs.

Top 50 candidate target genes of each of the 8 down-regulated miRNAs in ALS were also selected. Of the 400 target candidates identified for each of the 8 miRNAs, there was an overlap of 34 genes (Additional file 3: Table S3). These 34 genes have been reported to play a role in neurogenesis (ONECUT2 [69], KDM5A [70], NAT8L [71], NFIX [72]), mitochondrial regulation (IBA57 [66]) and autophagy (PRLA [73], SNCB [74], SOGA1 [54]). In addition, miR-3180-3p showed the most matching frequency (14 overlapped in 50 targets) in down-regulated miRNAs.

It is important to note that 3 genes (*IBA57*, *RAB3B* and *SOGA1*) were identified as candidate target genes of both up-regulated and down-regulated miRNAs (Additional file 2: Table S2, Additional file 3: Table S3). *IBA57* is known to be involved in the biosynthesis of mitochondrial [4Fe-4S] proteins [66]. Mutation of *IBA57* causes severe myopathy and encephalopathy [66]. Rab3B is a synaptic vesicle protein that interacts with the Rab3-interacting molecule isoforms as effector proteins in a GTP-dependent manner [64]. The search for an inhibitor of autophagy in the adiponectin signaling pathway led to the discovery of the Suppressor of Glucose from Autophagy (SOGA) [54]. These findings suggest that mitochondrial regulation, synaptic transmission and autophagy may be affected in ALS. Considering that the expression level of up-regulated miRNAs is higher than that of down-regulated miRNAs (Figure 2), these 3 genes may be down-regulated in the motor cortex of ALS.

### Gene ontology analysis of predicted target genes for disease-specific miRNAs

A total of 300 candidates for 6 up-regulated miRNAs and 400 candidates for 8 down-regulated miRNAs were nominated as targets for gene ontology enrichment analysis (biological process). Additional file 4: Table S4 (A, B) shows top 10 GO terms with MetaCore analysis and Additional file 4: Table S4 (C, D) shows top 10 GO terms with GOC analysis for standard GO terms. Two GO analyses suggested essentially similar biological process in up-regulated and down-regulated miRNA target genes.

The biological processes in ALS altered by these target genes were shown to be related to protein transport, synaptic vesicle-mediated transport, and localization for up-regulated miRNAs and nervous system development for down-regulated miRNAs, as shown in Additional file 4: Table S4.

### Decrease of AMBRA1 immunoreactivity in ALS

Since *SOGA1* was identified as one of the overlapped target genes predicted by 6 up-regulated and 8 down-regulated miRNAs, we hypothesized that alteration of autophagy is involved in the disease process of ALS. This may be supported by the findings that abnormal autophagy is involved in various neurodegenerative disorders, including ALS [75-79]. Therefore, we evaluated the protein expression levels of autophagy-related genes such as AMBRA1 [55], Beclin 1 [56], ULK1 [57] and ULK2 [57] by immunohistochemical examination of FFPE tissue.

**Figure 3:**
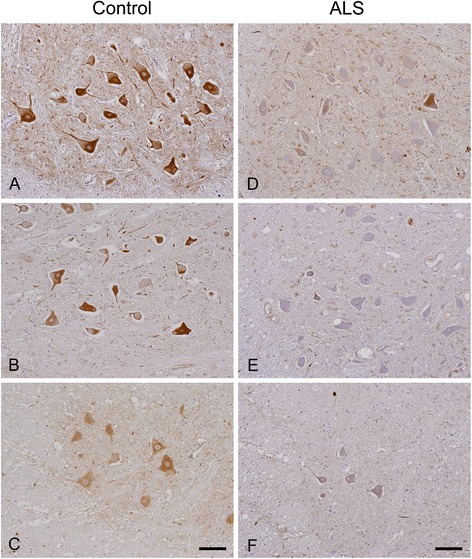
**AMBRA1 immunoreactivity in the anterior horn of the lumbar cord in 3 normal controls (A-C) and 3 cases of ALS (D-F).** AMBRA1 immunoreactivity is decreased in ALS relative to the controls. Bars = 100 μm.

In specimens from normal control subjects, anti-AMBRA1 antibody strongly immunolabeled the cytoplasm of upper and lower motor neurons in a diffuse granular pattern (Figure 3A-C), consistent with a previous study [80]. In ALS, AMBRA1 immunoreactivity was decreased in the majority of spinal anterior horn cells (Figure 3D-F), but not in the motor cortex (data not shown), in comparison with controls.

**Figure 4:**
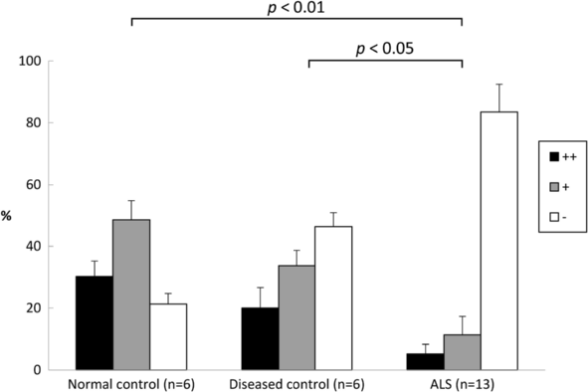
**AMBRA1 immunoreactivity in anterior horn cells from normal and diseased controls and patients with ALS.** The proportions of neurons showing intense/moderate (++, black column), weak (+, gray column), or no immunostaining for AMBRA1 (−, white column) relative to the total number of neurons in the anterior horn are indicated.

Semi-quantitative analysis of normal controls showed that 30.2% of anterior horn cells were moderately or intensely immunolabeled, 48.5% were weakly immunolabeled, and 21.3% were unstained (Figure 4). Similarly, in diseased controls, 20% of anterior horn cells were moderately or intensely immunolabeled and 33.7% were weakly immunolabeled (Figure 4). The differences in staining intensity between normal and diseased controls were not statistically significant. In ALS, a small proportion of anterior horn cells (5.2%) showed moderate or intense immunoreactivity, whereas the majority (83.5%) were unstained (Figure 4). The differences in staining intensity between normal control and ALS cases and between diseased control and ALS cases were significant.

Immunoreactivity for Beclin 1, ULK1 and ULK2 was observed in the neuronal cytoplasm in normal controls. No significant difference was found in the staining intensity of these proteins between ALS and normal controls (data not shown).

## Discussion

It has been reported that the formalin fixation and paraffin embedding process results in a marked reduction of detectable mRNA [81]. This process causes enzymatic degradation and chemical modification of RNA giving rise to cross-links with proteins and making RNA extraction difficult [52]. Therefore, a digestion step with proteinase K is required to eliminate cross-links and facilitate RNA extraction from FFPE samples [52]. The longer an RNA molecule is, the more likely cross-links will remain after proteinase K digestion, hence small RNA molecules will be easier to extract from FFPE samples and fragments larger than 200 nt will be harder to recover from FFPE samples. In addition, electrophoretic profiles of total RNA from frozen samples show three characteristic peaks: 40–80 nt, 90 nt and 150 nt, probably corresponding to tRNAs, rRNAs and snoRNAs, respectively. On the other hand, FFPE sample shows an accumulation of RNA fragments smaller than 200 nt [82]. These findings suggest that the amount of total RNA that can be extracted from a FFPE tissue sample is only a fraction of what can be extracted from its corresponding frozen tissue [81].

Although the number of subjects was small, we demonstrated for the first time that miRNAs extracted from FFPE samples of postmortem brain tissue from patients with neurodegenerative disorder (ALS) and neurologically normal controls were relatively well preserved; 12 of 26 samples (46.2%), for which the longest storage period was more than 7 years, provided RNA of sufficient quality. Thus, miRNAs appear to be relatively stable in FFPE samples, even those from postmortem specimens. It has been suggested that miRNAs are too small to be degraded [83]. However, this hypothesis has not been supported by any reported data. It is now known that active, mature miRNAs are processed and function via binding to Argonaute family proteins [84,85]. These protein-miRNA complexes may protect the functional population of miRNA from degradation, especially during the process of formalin fixation and storage in paraffin [49]. Peiró-Chova et al. [82] have shown that the quantity of miRNAs from FFPE samples was higher than that obtained from frozen samples, since degradation of total RNA can produce fragments in the small RNA size range that could cause an overestimation in the proportion of its small sized fragments.

ALS is characterized by loss of upper and lower motor neurons. TDP-43 is now known to be a major component of ubiquitinated inclusions in ALS and frontotemporal lobar degeneration with ubiquitinated inclusions (FTLD-U, since renamed FTLD-TDP) [86,87]. Thus, these neurodegenerative disorders comprise a new disease concept: “TDP-43 proteinopathy”. Up- or down-regulated miRNAs in ALS in the present study and previously reported results are shown in Additional file 5: Table S5. Williams et al. [36] compared miRNA expression in skeletal muscle of normal and ALS model mice (G93A-SOD1 transgenic mice) and demonstrated that miR-206, a skeletal muscle-specific miRNA in humans and mice, delays disease progression in SOD1 transgenic mice. miR-206 is up-regulated in the skeletal muscle of ALS patients [32]. Shioya et al. [19] studied the miRNA expression profile in frozen samples of frontal cortex from three ALS patients using microarray analysis and found that miR-29a, miR-29b and miR-338-3p were up-regulated. Up-regulation of miR-29b has also been reported in skeletal muscle of ALS patients [35]. De Felice et al. [33] evaluated miRNA expression in leukocytes obtained from ALS patients and healthy controls and demonstrated that miR-338-3p was also up-regulated in ALS. Seven miRNAs (miR-451, miR-1275, miR-328, miR-638, miR-149, miR-665 and miR-583) were also down-regulated in ALS. Importantly, in our present study, three miRNAs (miR-29a, miR-29b and miR-338-3p) were also up-regulated and four miRNAs (miR-328, miR-451, miR-638 and miR-665) were down-regulated in FFPE samples of the motor cortex in ALS. Recently, De Felice et al. [34] further demonstrated that miR-338-3p was over-expressed in leukocytes, serum, CSF and frozen samples of spinal cord in patients with ALS, and that miR-338-3p expression in leukocytes was correlated with disease duration, suggesting that miR-338-3p may be a relevant clinical biomarker of ALS. It is likely that some miRNAs are systemically dysregulated in ALS and that miRNAs remain stable even in FFPE postmortem samples that have been stored for a long period.

We further demonstrated that AMBRA1 was significantly down-regulated in the lower motor neurons in ALS. This is in line with the results of our miRNA analysis that AMBRA1 is most possibly regulated by miR-24-3p according to miRmap prediction (the rank 1 in canonical miRNAs of miRmap score = 97.37) and that miR-24-3p is one of the highly up-regulated miRNAs in ALS (Additional file 1: Table S1). AMBRA1 is widely expressed in neurons in the normal mouse brain and is localized to the endoplasmic reticulum, perinuclear cisternae and outer mitochondrial membrane [80]. AMBRA1 interacts with Beclin 1, promoting its binding to lipid kinase Vps34, thus mediating autophagosome nucleation [55]. AMBRA1 is also known to be a Parkin-binding protein involved in mitophagy [88]. Abnormal autophagy is involved in various neurodegenerative disorders, including Alzheimer’s disease [75,76], Parkinson’s disease [78], multiple system atrophy [79] and ALS [77]. A recent study has shown that administration of rapamycin, an MTOR-dependent autophagy activator, ameliorates neuronal degeneration in FTLD-TDP model mice [89]. By contrast, rapamycin also aggravates neuronal death in a mouse model of ALS [90]. Thus, induction or repression of autophagy should be taken into account when considering novel therapeutic approaches for TDP-43 proteinopathy.

## Conclusion

In conclusion, we have utilized FFPE brain samples from postmortem cases of ALS and neurologically normal controls and found that miRNAs extracted from these samples were relatively well preserved. Although further studies with a larger sample size are necessary, it is likely that archived FFPE postmortem samples can be a valuable source for miRNA profiling in neurodegenerative disorders.

## Additional files

Additional file 1: Table S1. Top 20 microRNAs up- or down-regulated in the motor cortex in ALS. Additional file 2: Table S2. Predicted target genes for ALS-specific up-regulated miRNAs identified by miRmap web-based open source software. Additional file 3: Table S3. Predicted target genes for ALS-specific down-regulated miRNAs identified by miRmap web-based open source software. Additional file 4: Table S4. Gene ontology enrichment analysis (bilogical process) of predicted target genes for disease-specific miRNAs. Additional file 5: Table S5. Summary of up- or down-regulated miRNAs in ALS.
